# Revisiting Ionic Liquid Structure-Property Relationship: A Critical Analysis

**DOI:** 10.3390/ijms21207745

**Published:** 2020-10-19

**Authors:** Wagner Silva, Marcileia Zanatta, Ana Sofia Ferreira, Marta C. Corvo, Eurico J. Cabrita

**Affiliations:** 1 UCIBIO, Chemistry Department, School of Science and Technology, NOVA University Lisbon, 2829-516 Caparica, Portugal; wm.silva@campus.fct.unl.pt (W.S.); asd.ferreira@fct.unl.pt (A.S.F.); 2 i3N|Cenimat, Materials Science Department, School of Science and Technology, NOVA University Lisbon, 2829-516 Caparica, Portugal; m.zanatta@fct.unl.pt (M.Z.); marta.corvo@fct.unl.pt (M.C.C.)

**Keywords:** ionic liquid, supramolecular organization, free volume, water, ion pair, physicochemical properties, transport properties

## Abstract

In the last few years, ionic liquids (ILs) have been the focus of extensive studies concerning the relationship between structure and properties and how this impacts their application. Despite a large number of studies, several topics remain controversial or not fully answered, such as: the existence of ion pairs, the concept of free volume and the effect of water and its implications in the modulation of ILs physicochemical properties. In this paper, we present a critical review of state-of-the-art literature regarding structure–property relationship of ILs, we re-examine analytical theories on the structure–property correlations and present new perspectives based on the existing data. The interrelation between transport properties (viscosity, diffusion, conductivity) of IL structure and free volume are analysed and discussed at a molecular level. In addition, we demonstrate how the analysis of microscopic features (particularly using NMR-derived data) can be used to explain and predict macroscopic properties, reaching new perspectives on the properties and application of ILs.

## 1. Introduction

**Figure 1:**
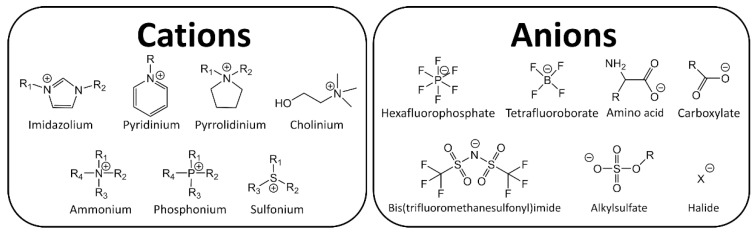
Typical ions in ionic liquids (ILs).

Ionic Liquids (ILs) are organic salts that melt commonly below 100 °C, therefore they are constituted entirely by charged species, usually an organic cation and an organic or inorganic anion. The extensive amount of possible combinations of known cations and anions (10^6^–10^18^) [1], results in different and unique physicochemical properties such as high thermal stability, large electrochemical window and low vapor pressure. Because of these attractive properties, ILs have garnered industrial and scientific interest, see Figure 1 [2,3].

The tunability and versatility of ILs have given rise to several applications such as solvents for synthesis and catalysis [4,5] CO_2_ capture and storage [6,7,8] energy generation and storage [9], extraction/dissolution of biomass [10,11], and active pharmaceutical ingredients [12,13].

**Figure 2:**
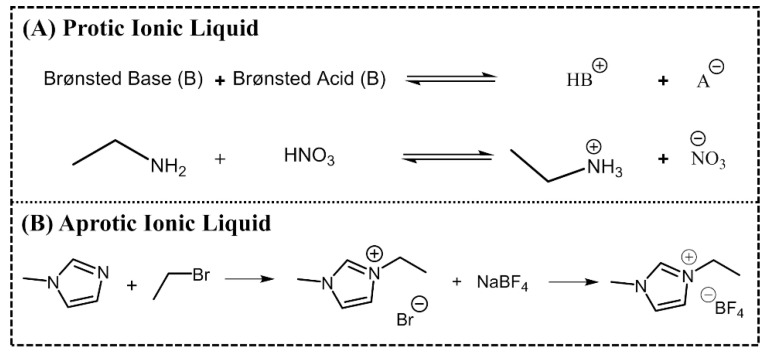
Typical synthetic route of a protic ionic liquid (PIL) (**A**) ethylammonium nitrate and an APIL (**B**) 1-ethyl-3-methylimidazolium tetrafluoroborate.

ILs are normally divided into two classes: aprotic ionic liquids (APILs) and protic ionic liquids (PILs). PILs are formed by the transfer of protons from Brønsted acid to Brønsted base, this proton transfer does not take place in AILS due to the nature of the ions constituting the salt. PILs are generally prepared through a neutralization reaction and APILs by a quaternization reaction followed by anion exchange, Figure 2 [14,15].

**Figure 3:**
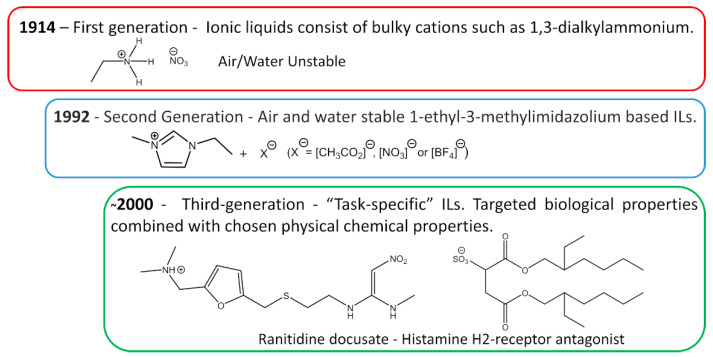
Generations of ILs through time.

The first IL reported in the literature is a controversial matter. Most authors state that the history starts with Paul Walden’s discovery in 1914 with the report of ethylammonium nitrate ([EtNH_3_][NO_3_]) synthesis, see Figure 3 [16]; however, some authors attribute the first IL to Gabriel and Weiner in 1888 [17], with the synthesis of ethanolammonium nitrate. This confusion arises from the reported melting point, while Walden’s compound was characterized with a melting point of 13–14 °C, Gabriel’s compound exhibited a melting point of 50 °C, substantially higher than room temperature. These works marked the beginning of the First Generation of ILs. Cations of the first generation of ILs are characterized by large volumes, such as 1,3-dialkyl-imidazolium or 1-alkylpyridinium and anions based mostly on halogen aluminate (Al^+3^) [1,18]. The disadvantage of this generation was the instability in relation to air and water.

The introduction of the *Second Generation* of ILs comes with the preparation of air and water stable ILs, reported in 1992 by Wilkes and Zaworotko [19], based on the 1-ethyl-3-methylimidazolium cation and alternative anions, [CH_3_CO_2_]^−^, [NO_3_]^−^ and [BF_4_]^−^, see Figure 3. ILs from the second generation are much easier to handle than those of the first generation. It was due to this fact and their particular properties, that IL-based research has become one of the major scientific topics over the last 25 years [20].

During the first decade after the publication of Wilkes and Zaworotko [19], solid scientific background was built on the subject of ILs and specific applications started to be targeted. In Ann Visser’s paper, a series of functionalized imidazolium-based ILs was synthesized to extract heavy metals, Hg^+2^ and Cd^+2^ from aqueous solutions [21,22], and the terminology “Task-Specific” was introduced in connection to IL applications. Tuneable physical and chemical properties according to the desired application are what defines the Third Generation of ILs, see Figure 3. Among these properties, rationally selected biological action is already a reality, as evidenced by Hough’s review [23] where several ILs candidates were used as Active Pharmaceutical Ingredients (API) or precursors.

As ILs applications matured, a detailed understanding and predictive capabilities towards their thermophysical properties have become progressively more important.

Since the introduction of the second generation of ILs, this class of compounds was brought to trend topics in the scientific world, and several works correlating structure and properties have been published. However, many issues are still the subject of intensive investigation, such as the existence of ion pairs, the definition of ionicity, the structuring of water inside the ILs and the mechanism of diffusion, and mainly the relationship between the free volume and transport properties. Therefore, this review presents a rationalization of IL properties starting from the classical chemical structure-property relationship generally described in the literature, followed by a detailed discussion analysing the reasons for their properties, starting from the smaller scale towards higher degrees of complexity, i.e., going from the microstructures to higher organized aggregates and solvent effect. The physicochemical properties of the second generation of ILs are revised and correlated to free volume theory for the first time. In addition, the application of NMR to determine these properties is highlighted.

## 2. Structure–Property Relationships 

The widespread application of ILs has motivated the need for a detailed understanding of these materials, to rationalize and predict their behaviour. Their tunability reinforces the necessity for a predictive capability towards their thermophysical properties and motivates the efforts to resolve the molecular-scale details of structure, dynamics, and interactions in ILs. Several reviews on structure–property relationships for ILs fundamental thermal and physicochemical bulk properties can be found in the literature [24,25,26,27]. The predictive methodologies generically consider either empirical or theoretical approaches for representing properties such as density, surface tension, sound velocity, heat capacity, melting points, and electrical conductivity [28,29,30,31].

Empirical approaches correlate IL properties with their respective structure. Among the transport properties of ILs, viscosity is one of the most studied because of its importance to electrochemical and separating applications. IL viscosities usually vary between 20 and 40,000 cP, which is 1 order of magnitude higher than that of conventional solvents, such as water, with a viscosity of ~1 cP. A general trend is that the viscosity decreases with the increasing size of ions, with a greater dependence on the anion. For cations, the viscosity increases with the increasing length of the alkyl chains partially due to stronger van der Waals (VDW) interactions. A relationship between the viscosity and the symmetry of the cations was also disclosed, with asymmetric cations exhibiting linear correlations opposing the small and symmetric cations. Besides the size and shape of ions, viscosity is also closely related to intermolecular interactions. High interaction energy, due to electrostatic forces and VDW interactions between anion and cation, leads to higher viscosity. Additionally, viscosity also correlates with ion stacking caused by hydrogen bonding, with smaller interactions leading to lower viscosity, and with the delocalization of the charge on the anion that weakens the hydrogen bond, and consequently decreases the viscosity. Conductivity is also frequently studied and lays within the range of 0.1–30 m.S cm^−1^. At low temperatures, the structures of cations and anions influence the conductivity of ILs. The conductivity usually decreases with the increasing length of alkyl chains of cations, and the anions have a more significant effect on the conductivity, with a higher influence of the charge distribution and the number of charge carriers [32].

**Figure 4:**
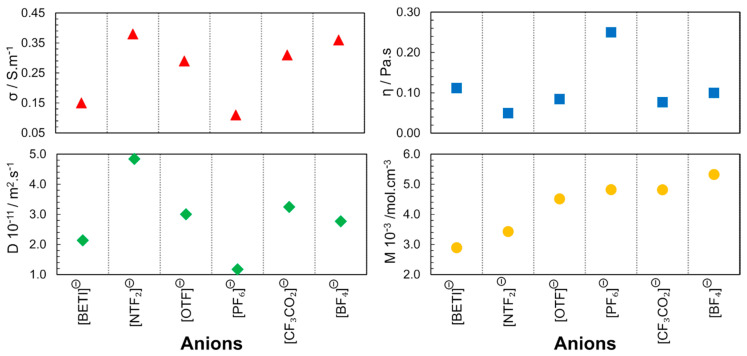
Anion dependence of electrical conductivity (*σ*), viscosity (*η*), self-diffusion (*D*) and molar concentration (*M*) for [C_4_mim][X] at 298K and 0.1MPa. The diffusion coefficient presented is the result of the sum of cation and anion self-diffusion. Image created using data published in references [33,36].

The group led by Watanabe from Yokohama National University [33,34,35,36], published a series of articles with some of the first correlations between IL structure and properties. These publications highlighted the relationship between the ionic diffusivity, viscosity, and molar conductivity for different cationic and anionic structures. The first publication in 2004 [33] reports the study of the 1-butyl-3-methylimidazolium ([C_4_mim]^+^) cation with different fluorinated anions ([NTF_2_]^−^, [OTF]^−^, [PF_6_]^−^, [CF_3_CO_2_]^−^, [BF_4_]^−^). Through self-diffusion and conductivity experiments, it was found that the high rate of ions contributing to the ionic conductivity within the diffusion component is related to the poor interaction of the anion with the cation. The electronegative fluorine atoms and perfluorosulfonyl groups in the anion contribute to the distribution of the anionic charge in phosphate, borate and imide, respectively. This study concerned only anion effects. In this way, diffusive species in the IL that contribute to the ionic conduction depend only on the anion character. With these results, it is evident that the size, shape and geometry of the anion determine the strength of the electrostatic interaction between the cation and the anion (Figure 4). Moreover, the proximity between the ions also enables hydrogen bonds between the fluorine atoms of the anion and the cation protons. These factors justify the higher viscosity and lower conductivity observed for [C_4_mim][PF_6_]. 

**Figure 5:**
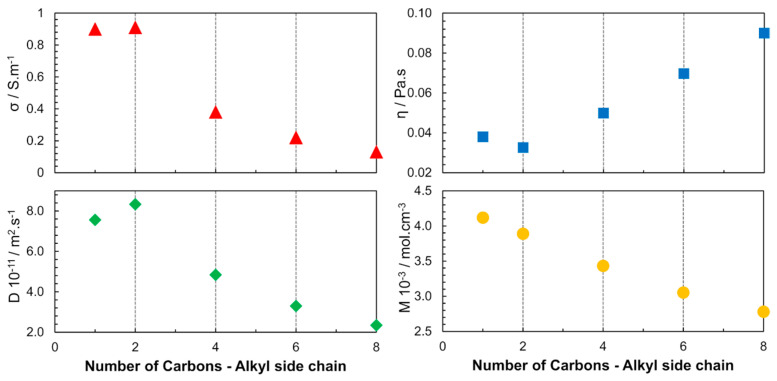
Alkyl-chain-length dependence of viscosity (η), electrical conductivity (σ), self-diffusion (D) and molar concentration (M) for [C_n_mim][NTF_2_] at 298 K and 0.1 MPa. The diffusion coefficient presented is the result of the sum of cation and anion self-diffusion. Image created using data published in references [34,36].

In 2005, another publication focused on the cation structure, where the alkyl chain length of 1-alkyl-3-methylimidazolium bis(trifluoromethane sulfonyl)imide ([C_n_mim][NTF_2_]) was varied (*n* = 1, 2, 4, 6, 8) to study the ILs properties over a wide temperature range [34]. The increase in the alkyl chain length was found to cause changes in the interaction forces (VDW). The properties of the ILs were determined by the cumulative effect of the electrostatic interaction between the ionic species and the induction interactions between the ions, aggregates, and clusters (Figure 5). Among the studied ILs, [C_2_mim][NTF_2_] had the highest diffusivity and electrical conductivity and also the lowest viscosity at 298 K and 0.1 MPa.

**Figure 6:**
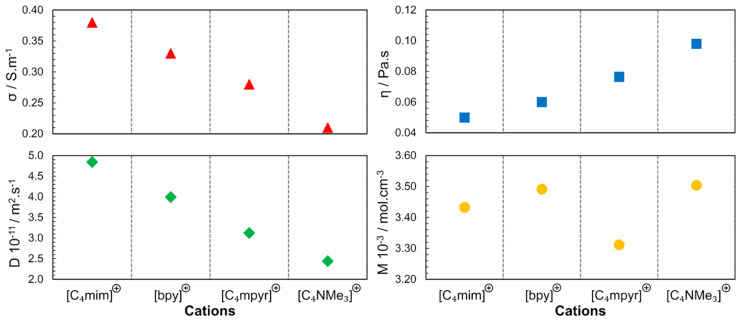
Cation dependence of viscosity (*η*), electrical conductivity (*σ*), self-diffusion (*D*) and molar concentration (*M*) for [NTF_2_]^−^ -based ILs at 298 K and 0.1 MPa. The diffusion coefficient presented is the result of the sum of cation and anion self-diffusion. Image created using data published in references [35,36,37].

In 2006, different classes of cations were studied using 1-butyl-3-methylimidazolium ([C_4_mim]), *N*-butylpyridinium ([bpy]), *N*-butyl-*N*-methylpyrrolidinium ([C_4_mpyr]^+^) and *N*-butyl-*N*,*N*,*N*,-trimethylammonium ([C_4_NMe_3_]^+^) combined with [NTF_2_]^−^ anions [35]. As depicted in Figure 6, the molar concentrations (***M*** in mol.L^−1^; in neat ILs, the molar concentration can be obtained by the ratio between density and molar mass of pure IL) of the studied ILs are close, leading to the conclusion that the steric hindrance is another factor that determines the properties of the ILs, not only due to the geometry but also due to the rotational dynamics of the substituents in the cationic families. Accessibility to the charged center (region with the higher charge density) determines the strength of the interaction between the cation and the anion. In these results, also implicit is the dependence of the physicochemical properties from the molecular dynamics; the speed of molecular rotation and the rotation of its functional groups is a factor that determines the proximity of the neighbouring species. The following order was found for the diffusion coefficient and conductivity: [C_4_mim]^+^ > [bpy]^+^ > [C_4_mpyr]^+^ > [C_4_NMe_3_]^+^.

The nature of the ionic species (size, shape, geometry, molecular dynamics and mass) and the interactions (cation–cation, cation–anion, anion–anion) that take place between them determine the macroscopic properties of the bulk ILs, that is, to understand the physical properties such as viscosity, density, electrical conductivity, surface tension, refractive index, melting point, among others it is necessary to look at the (sub) microscopic level.

## 3. Structural Organization 

The local nanostructural organization and the physicochemical properties of ILs are directly related to intermolecular interactions present in the system. The non-directional (coulombic) interactions lead to a more isotropic distribution of ionic species and the directional interactions (Van der Waals and hydrogen bonding) to an anisotropic distribution. An intermolecular interaction study based on Density Functional Theory (DFT) using [C_2_mim][BF_4_] and [C_4_mim][PF_6_] ILs demonstrated that the Coulombic Force contributes with 70% of total energy, which indicates that the other interactions, like hydrogen bonding, π–π stacking and dispersion interactions, should also affect significantly the physicochemical properties [38,39]. Recently, a thorough review focused on understanding heterogeneous microstructures and dynamics of ILs in bulk liquids, in mixtures with cosolvents, and in interfacial regions was published highlighting the importance of the interplay among the intra- and intermolecular interactions [40].

**Figure 7:**
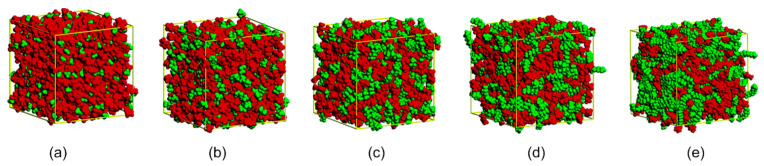
Snapshots of simulation boxes of [C_n_mim][PF_6_]. Red=polar and green = nonpolar. (**a**) [C_2_mim][PF_6_]; (**b**) [C_4_mim][PF_6_]; (**c**) [C_6_mim][PF_6_]; (**d**) [C_8_mim][PF_6_] and (**e**) [C_12_mim][PF_6_]. Figure adapted from [45].

Cationic species based on imidazolium ([C_n_mim]^+^) structures present polar (imidazole ring) and nonpolar (alkyl chain) domains. One of the first reports on the presence of nanosegregated domains in ILs associated the aggregation behaviour to the cation’s side-chain length by multiscale coarse-graining method [41] a heterogeneity order parameter concept that quantifies the geometric heterogeneity in IL was later introduced [42,43,44]. Since then, molecular dynamics (MD) simulation and X-ray analysis of imidazolium ILs (ImIL) have been extensively used to observe the nanoscale IL structure, indicating a sophisticated special organization in the polar domain with a 3D network of ionic channels. This supramolecular organization remains practically unchanged in the solid and liquid phase [45,46,47,48,49,50,51,52]. Structural heterogeneity is observed with increased alkyl chain size as a consequence of the segregation of non-polar domains (Figure 7); the electrostatic interactions between charged sites assist the formation of polar domains and the uncharged groups are driven out of this region [25,45].

The main structure of ImILs can be described as a supramolecular polymeric network of intermolecular interactions that determine the interionic distances and consequently the existence of ion pairs and/or aggregates and free volume. 

### 3.1. Ion Pairs

The concept of IP in ILs has been discussed and revised many times in the literature; for this reason, it will be only succinctly explored here.

The phenomenon of ion pair formation is of fundamental importance for different application fields, such as: (i) catalysis—due to its influence on reaction rates or transfer of chirality [53,54]; (ii) CO_2_ capture—being decisive on sorption mechanism [55,56]; (iii) ion pair chromatography or selective ion electrodes [57,58]. Since ILs are composed entirely of ions, a higher value of conductivity is expected. However, through experimental data, a smaller conductivity of the neat IL, when compared with diluted systems, has been reported [59,60,61,62]. This phenomenon has been interpreted as an indication of the existence of ion-pairing [63], transfer of charge [64] or the formation of small clusters of ions [65]. Marcus and Hefter defined ion pairing as:“… (partial) association of oppositely charged ions in electrolyte solutions to form distinct chemical species called ion pairs” [63].

To evaluate the conductivity and understand how ionic an IL is, the concept of ionicity (I) was proposed by Watanabe and co-workers [33,34,35,36,66]. For this purpose, the molar conductivity ratio ($\frac{\Lambda_{imp}}{\Lambda_{NMR}}$) of an IL is used as a measure of the ionicity: $I=\frac{\Lambda_{imp}}{\Lambda_{NMR}}$. $\Lambda_{imp}$ is the ionic conductivity measured by the electrochemical impedance method and $\Lambda_{NMR}$ is calculated from the self-diffusion coefficients of cations (D^+^) and anions (D^−^), determined from pulse-field-gradient spin–echo (PGSE) NMR, using the Nernst–Einstein Equation (1).

(1) $$\Lambda_{NMR}=\frac{N_{A}*e^{2}}{kT}\left(D^{+}+D^{-}\right)$$

where $N_{A}$ is the Avogadro number, e is the electric charge on each ionic carrier, k is the Boltzmann constant, and T is the absolute temperature. 

The molar conductivity ratio indicates the proportion of ions that contributes to ionic conduction from all the diffusing species and therefore can be used as a quantitative measure of ionicity.

Another important feature of ILs to be considered for a tailored application is their polarity. However, due to the difficulty of establishing a polarity scale for ILs the hydrogen-bond basicity (β) (a Kamlet–Taft parameter) has been studied. To estimate the hydrogen-bonding interaction energy in the equimolar cation–anion mixture (*E*_HB_ (kJ.mol^−1^), the COnductor-like Screening MOdel for Real Solvents (COSMO-RS) has been used for several ILs [67].

From a qualitative perspective, ionicity and hydrogen-bonding interaction energy should be related, since it is expected that ILs with a lower *E*_HB_ (stronger hydrogen-bonding interaction) have a lower ionicity.

**Figure 8:**
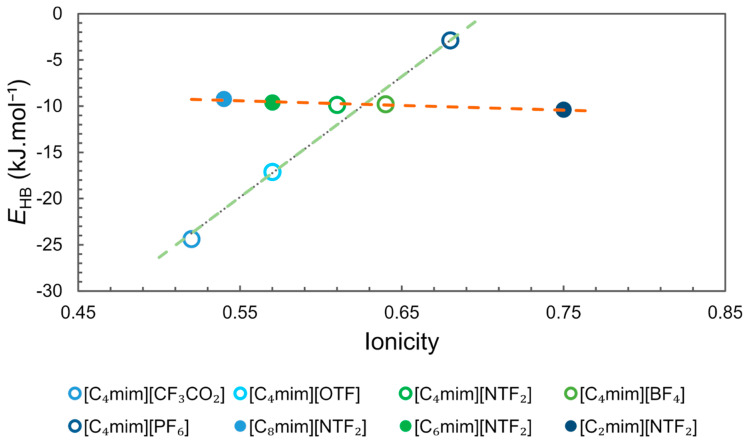
Ionicity ($\frac{\Lambda_{imp}}{\Lambda_{NMR}}$) versus hydrogen-bonding interaction energy (*E*_HB_). Ionicity data from Ueno et al. [66] and *E*_HB_ data from Cláudio et al. [67].

The values of ionicity of some ILs reported by Ueno et al. [66] and the *E*_HB_ reported by Cláudio et al. [67] were compiled and organized in Figure 8. The analysis of the data clearly shows two trends, one for ILs with the same cation [C_4_mim][X] (R^2^ 0.97) and another for ILs with the same anion [C_n_mim][NTF_2_] (R^2^ 0.93). For the first group, ionicity and *E*_HB_ are strongly correlated and ionicity increases with the decrease in the strength of the hydrogen-bonding interaction (higher *E*_HB_) that is being controlled by the nature of the anion. For the second group, an increase in ionicity can be observed with the decrease in the size of the imidazolium side chain, but the interaction energy *E*_HB_ remains almost unchanged. These observations indicate that ionicity is influenced by other factors than the strength of the interaction between cation and anion by hydrogen bonding. For the first group the ionicity sequence for the ILs studied follows the reported basicity scale, [CF_3_CO_2_]^−^ > [OTF]^−^ > [BF_4_]^−^ > [NTF_2_]^−^ > [PF_6_]^−^ [67,68]. For the same cation, the stronger the interaction with the corresponding anion by hydrogen bonding, the lower the ionicity. In the second group, the behaviour observed for ILs with the same anion could be correlated to the structural heterogeneity of ILs, which increases according to the size of the cation side chain, as shown in the MD simulations (Figure 7) [45]. Segregation of non-polar domains leads to aggregate formation, which reduces the conductivity and ionicity of ILs. 

According to Equation (1), the ionicity data of Figure 8 show that not all the ions that are diffusing contribute to the conductivity, since generally, the values are non-integer and smaller than expected. To account for this the theory of ionicity in ILs was reformulated by Hollóczki et al. with the contribution of the *Charge Transfer* concept [64], closing a gap identified in the previous theory [66]. Considering that the value of the partial charge is a non-integer value, normally lower than expected due to the transfer of charges through hydrogen bonds, it has been estimated that the effective charge is reduced by a factor of ±0.8, resulting in smaller conductivity [64,66]. Another possible explanation is that the concentration of “free” ions is reduced by the formation of ion pairs (neutral charge) or larger clusters leading to the reduction of conductivity [65].

**Figure 9:**
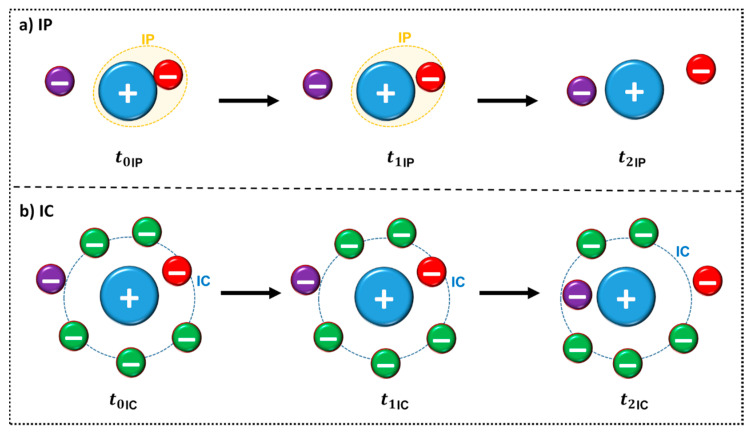
Schematic representation of the definition of an ion pair IP (**a**) and ion cage (IC) (**b**) adapted from reference [72]. The ion representations are merely illustrative, IL ions are not simple spheres, they have more complex shapes that assign directionality to specific regions of interaction.

For the existence of ion pairs to be responsible for changes in physicochemical properties, such as the conductivity, the ion pair must be long-lived enough to be detected, i.e., longer than the time required for thermal motion to cause the ions to move as separate species [63]. The lifetime of the ion pair can be determined through MD simulations and varies according to the choice of the reference distance used in the calculation, presenting values between nanoseconds to picoseconds [69,70,71]. Zhang and Maginn studied the lifetime of 29 ILs through MD [72]. They used a previously established definition of ion pair (IP) and ion cage (IC) [63,65] (Figure 9), to quantify exchange between the counterions. IP can be defined as a partial association of oppositely charged ions to form distinct chemical species called IP (Figure 9a), whereas IC is formed by all the anions in the first solvation shell of a cation or vice versa, (Figure 9b). The authors also state that “*the concept of a physical ion pair should not be taken too literally*” [72]. From the figure it is possible to infer that: (a) At a given time *t*_0_, an IP is defined by the anion (red) with the shortest distance from a central cation (blue) or vice versa. The dashed orange line represents the first IP contact. This IP remains formed at a later time *t*_1_ until another counterion (purple) comes closer to the central ion at time *t*_2_ as observed in Figure 9a. A similar observation can be made from Figure 9b for IC structure: at a given time *t*_0_, an IC is formed by all of the anions in the first solvation shell (dashed blue line) of a cation or vice versa. An IC remains formed later (*t*_1_) if no counterions leave or enter the first solvation shell. Otherwise, if either a counterion leaves (indicated in red) or a new counterion enters (indicated in purple), which happens at time *t*_2_, the IC is considered broken. In addition, it is possible to punctuate that the *t*_1*IC*_ is larger than *t*_1*IP*_ [72].

Zhang et al. [72] reported a strong linear correlation that crosses the origin when plotting the average self-diffusivities ($D=\;\left(D^{+}+\;D^{-}\right)/2$) against the inverse of IP and IC lifetimes. Even more surprising, this correlation is independent of the temperature or nature of IL. In addition, the authors demonstrated that long lifetimes for a particular IL lead to low diffusivity and consequently high viscosity. This means that the transport properties are strongly influenced by the rate of formation/disruption of the IPs and ICs.

**Figure 10:**
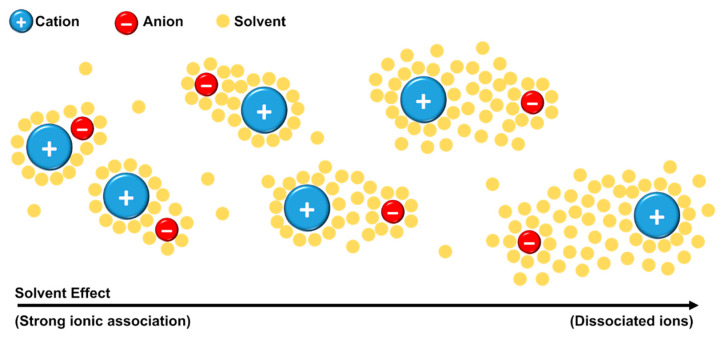
Schematic representation of a generic IL in solution—Effect of solvation shells on ionic association.

Kirchner and co-authors [69] defined that the existence of ion pairs should be considered only if the lifetime of the contact between the ions is long enough for the unit to travel (at least) the distance of its own size. Through MD simulations, no evidence of ion pair formation was found. The group explained that, since the contact between the ions occurs in a few picoseconds, this time is not enough for the unit to travel the distance of its own diameter. The authors pointed out that “ion pair” as a neutral entity is a misinterpretation. When the ions are close to each other, they used the terminology “ionic association” instead of “ion pair”. Even with the discordance of ion-pair definition, a point of general accordance is that the structural organization and molecular dynamics of the ILs in solution depend on solution concentration and the nature of solvent [69]. The solvent can collaborate in the formation of ion pairs (or ionic association) or it can solvate the ions making it difficult for the ions to approach each other, Figure 10 [73,74].

Despite the use of MD simulations, nuclear Overhauser effect (NOE) NMR spectroscopy has also been described as an alternative method for the systematic study of the interactions between ions in ILs systems. While the NOE correlation is normally used for studying local structure in liquids and solutions results from short-range effects (d < 5 Å), Gabl et al. [75] have shown that in ILs this is not necessarily true. They have demonstrated that due to the high viscosity of IL systems, the experimental cross-relaxation rate giving rise to the NOE can give information on the mutual position of interacting species far beyond the first coordination shell. Therefore, the information provided by NOE analysis allows the observation of ion organization over longer distances rather than just the local structure [76,77]. 

Hu et al. [78] have used DFT calculations with PCM (polarizable continuum model) to account for the effect of different solvents on the on the anion–cation and ion–solvent interactions. They found that the binding energies of the anion–cation obtained in solvents are quite different from that obtained in gas phase calculation, due to the remarkable solvation energy difference between the contacted ion pair and separated anion/cation. Their results give a theoretic reason why the anion–cation prefer to form contact ion pairs in solvents with low dielectric constant, whereas a high dielectric constant solvent has a great ability to separate the anion–cation.

In summary, ion-pairing in ILs, or more specifically, the lifetime of ion-pairing affects properties such as ionic conductivity and diffusivity, which will consequently affect the designed application.

### 3.2. Free Volume 

#### 3.2.1. Free Volume Theory and Transport Properties

The conductivity (σ), viscosity (η) and self-diffusion (*D*) are the main transport properties of ILs (mass transport) and are critical aspects of the overall properties and applications. In ILs, the transport properties are determined by conditions such as temperature and pressure and the nature of its counterions, more precisely the calculated molecular volume of the ion pair ($V_{IonPair}$) (Equation (2)), obtained by the sum of the molecular volume of each ion, cation ($V_{ion}C^{+})$ and anion $\;\left(V_{ion}A^{-}\right)$ [79].

(2) $$V_{IonPair}=V_{ion}\left(C^{+}\right)+V_{ion}\left(A^{-}\right)$$

**Figure 11:**
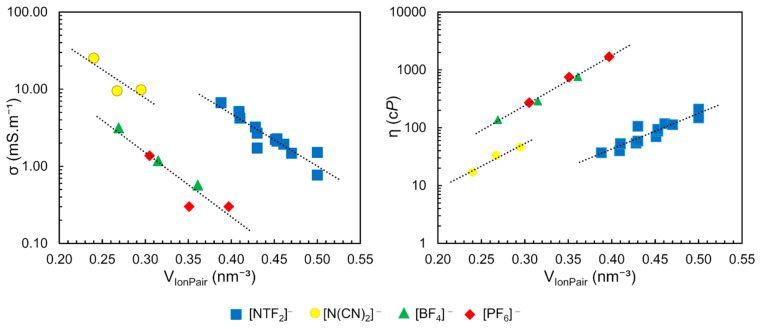
Conductivity and viscosity dependence of molecular volume of ion pairs for ILs present in Table 1. Conductivity acquired at 295 K and viscosity at 294 K. Each dotted line represents the correlation for a different group of IL with same anion nature. Figure adapted from reference [79].

Based on the free volume models of liquids, correlations between transport properties and molecular volume are qualitatively expected. In this context, Slattery et al. have shown a strong correlation of $V_{IonPair}$ (Table 1) with viscosity and conductivity (Figure 11) [79]. The conductivity of these ILs decreases exponentially with increasing $V_{IonPair}$ (a) while the viscosity increases (b). The authors found three separated clusters in these correlations, according to the anion type: [BF_4_]^−^ and [PF_6_]^−^; dicyanamide [DCA]^−^ and [NTF_2_]^−^, as observed in Figure 11

Similar dependence was observed between the density (ρ) and the nature of the anion. In the same work, the authors demonstrated a relationship between the molar concentration (M) of neat ILs, obtained by the ratio between density and molar mass of pure IL (in mol.L^−1^) as previously described, with the $V_{IonPair}$, resulting in an exponential correlation ($M=a\times {V_{IonPair}}^{-b}$) with R^2^ = 0.9889. These results showed the possibility to predict the density, viscosity and conductivity as a function of the molar concentration (M) and the anion type, with optimum correlation values (R^2^ of 0.9889, 0.9976 and 0.9871 respectively), using the data in Figure 11 and Table 1 [79,80].

Taking into account that the transport properties demonstrated above, strongly depend on free volume, the study of this concept is of fundamental importance to design the properties of materials. The theory of the free volume effect can provide insight into gas solubility phenomena in IL and consequently resolve catalytic activity issues [81].

According to the free volume theory, the total volume of a liquid ($V_{l}$) consists of two components: occupied ($V_{occ}$) and free volume ($V_{f}$), Equation (3) [82].

(3) $$V_{l}=V_{occ}+V_{f}$$

**Figure 12:**
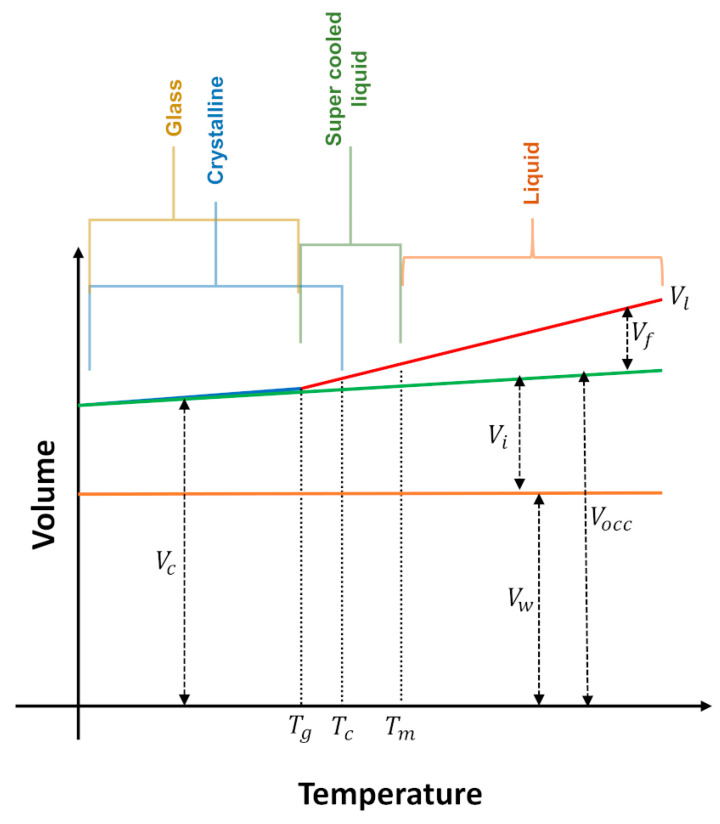
Schematic representation of the constitution of the volume, at isobaric condition. $V_{i}$ interstitial volume; $V_{f}$ free volume; $V_{l}$ total volume of the liquid; $V_{c}$ volume of the crystal; $V_{W}$ Van der Walls volume; $V_{occ}$ occupied volume; $T_{g}$ glass transition temperature; $T_{c}$ —crystallization temperature; $T_{m}$ Melting temperature. Adapted from references [3,82].

The occupied volume is assumed to be incompressible while the $V_{f}$ is compressible. $V_{occ}$ can be defined as in Equation (4).

(4) $$V_{occ}=V_{i}+V_{w}$$

where $V_{i}$ means interstitial volume (a free volume existent even in the crystalline state), and $V_{w}$ represent the Van der Waals volume (related to the minimal volume occupied by molecules impenetrable for other molecules at ordinary temperatures). Based on temperature-dependent crystal structures, a radii-based methodology can be applied for the calculation of $V_{w}$ of organic salts. In this approach the atoms are assumed as fused spheres with Van der Waals radii and the space occupied by the hard spheres is calculated [83]. The schematic representation of the constitution of the volume of a liquid, glass or crystal according to temperature is illustrated in Figure 12 [3].

From Figure 12 it follows that when a given IL at a temperature above its melting point ($T_{m}$) is cooled, its volume ($V_{l}$) decreases until it reaches the crystallization temperature ($T_{c}$) (liquids with maximum density, like water, do not obey this behavior). $T_{c}$ may coincide with $T_{m}$, but normally the liquid remains in the supercooled condition and crystallizes. Some liquids do not crystallize, instead, they show a transition to the glass state at $T_{g}$. The decrease in $V_{l}\;$ can be almost entirely accounted for by a decrease in the free volume, $V_{f}.$ A similar trend is observed for the volume of the crystal ($V_{c}$), that also changes with temperature, although to a lesser extent, because $V_{i}$ has a smaller expansion coefficient than the $V_{f}$. Assuming that perfect single crystals are formed, $V_{c}$ is very well represented by the molecular volume (this is only valid for the single crystalline state).

The overall $V_{f}\;$ is distributed locally into cavities, voids or holes ($V_{h}$). The local free volume, $V_{h}$, consists therefore of irregular shaped holes and cavities formed by a particular molecular distribution. The holes are formed by the coalescence of other free space regions and have random size, shape and location. These characteristics are constantly changing due to the displacement of the molecules into the bulk liquid. Given the relationship between free volume and transport properties such as viscosity and conductivity, the study of free volume in ILs and how it may influence its physicochemical properties provides a link between the structures of ILs and its effects for a particular application [3,84].

The density dependence of pressure–temperature can be used to estimate $V_{f}$ using the Sanchez–Lacombe (SL) equation of state (EoS), Equation (5). SL theory introduces holes to explain variations on compressibility and density (isobaric expansivity ($\alpha_{p})$ and isothermal compressibility ($K_{T}$)) [85,86].

(5) $$\begin{array}{c} {\tilde{\rho }}^{2}+\tilde{P}+\tilde{T}\left[ln\left(1-\tilde{\rho }\right)+\left(1-\frac{1}{r}\right)\tilde{\rho }\right]=0\\ \tilde{T}\equiv \frac{T}{{T\;}^{*}}\;\tilde{P}\equiv \frac{P}{{P\;}^{*}}\;\tilde{\rho }\equiv \frac{\rho }{{\rho \;}^{*}}\\ {\varepsilon \;}^{*}=R{T\;}^{*}\;{v\;}^{*}=R{T\;}^{*}/{P\;}^{*}\;r=\frac{M_{w}{P\;}^{*}}{R{T\;}^{*}{\rho \;}^{*}} \end{array}$$

where ${P\;}^{*}$, ${\rho \;}^{*}$ and ${T\;}^{*}$ represent characteristic parameters of the SL equation; $M_{w}$ is the molecular weight; R is the gas constant. A pure fluid is completely characterized by three molecular parameters in this EoS: ${\varepsilon \;}^{*}$ segment interaction energy, r segment number of one molecule and ${v\;}^{*}$ segment volume. ${v\;}^{*}\times r$ is equal to the hard-core volume. The $V_{f}$ can be calculated as shown in Equation (6), where $V_{m}$ is the molar volume, $M_{w}$ is the respective IL molecular weight and ρ is the density.

(6) $$V_{f}=\;V_{m}-\left({v\;}^{*}\times r\right);\;V_{m}=\frac{M_{w}}{\rho }$$

Analysing Equations (5) and (6), it is possible to observe that $V_{f}$ increases with temperature and decreases with pressure. When the temperature increases (at isobaric conditions), the kinetic energy of molecules and the total space between molecules increases; modifying the force between atoms and, consequently, the physicochemical properties [87,88,89,90].

**Figure 13:**
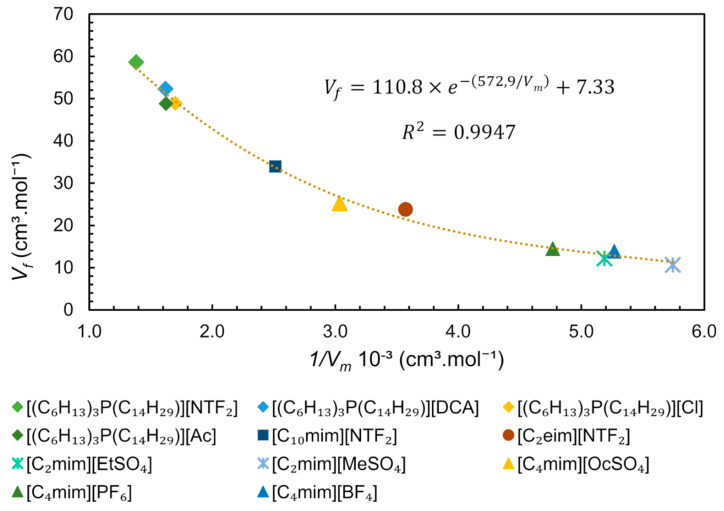
Free Volume ($V_{f}$) dependence of inverse of Molar Volume ($1/V_{m}$ ) of ILs at 313 K and 0.1 MPa. The data for trihexyl(tetradecyl)phosphonium-based ILs from references [87,88], [C_4_mim]^+^-based ILs from [89] and the remaining ILs from [90].

Since the free volume and molar volume are correlated (Equation (6)), we performed an analytical treatment of literature data, plotting $V_{f}$ values (estimated by SL EoS) versus the inverse of $V_{m}$ for some ILs obtained at 313 K and 0.1 MPa, as shown in Figure 13. From this data treatment, it is possible to observe a strong exponential correlation between free volume and molar volume, represented by the equation $V_{f}=110.8\times \left(e^{-572.9/V_{m}}\right)+7.33$. This equation is very useful for predicting the free volume of pure ILs at studied conditions, being dependent only on density (or molar concentration), which in turn is dependent on temperature and pressure. The molar concentration at a given pressure and temperature is easily determined by reorganizing the Equation (6) ($M=\frac{\rho }{M_{W}}$) if the density of an IL under such specific conditions is available, in other words, 1/$V_{m}$, is equal to molar concentration (*M*).

The ILs constituted by the trihexyltetradecylphosphonium [(C_6_H_13_)_3_P(C_14_H_29_)]^+^ family have a large free volume compared to ImIL (Figure 13). These cations have very high molecular volume and molecular mass and are less packed, allowing mobility and providing “available space” to accommodate anions. For this particular cation ([(C_6_H_13_)_3_P(C_14_H_29_)]^+^) it is observed that the combination with anions [DCA]^−^, [Cl]^−^ and [Ac]^−^ has similar free volume and practically the same density at the studied conditions, ~0.88 g.cm^-3^. However, the anion [NTF_2_]^−^, which has a molecular mass larger than its analogues, leads to higher density (1.06 g.cm^−^^3^) and a lower molar concentration, indicating that molecular mass is the determinant for the free volume. The IL [C_2_mim][MeSO_4_] presents one of the highest densities (1.28 g.cm^−^^3^) and the smaller free volume between the studied ILs (Figure 13), indicating that the counterions are quite packed. The free volume can be related to ILs applications/properties such as gas sorption capacity and solubility of compounds. From Figure 13, it is possible to observe a higher free volume for phosphonium ILs than ImILs, suggesting that this free space is the reason for the higher CO_2_ sorption capacity observed in phosphonium-based ILs using non-basic anion as counterion [81]. 

At this point, it is possible to state that the molecular structural organization determines the free volume. This free volume can be evaluated as a free volume fraction $(F_{fv}$) of a given IL as in Equation (7).

(7) $$F_{fv}=V_{f}/V_{m}$$

**Figure 14:**
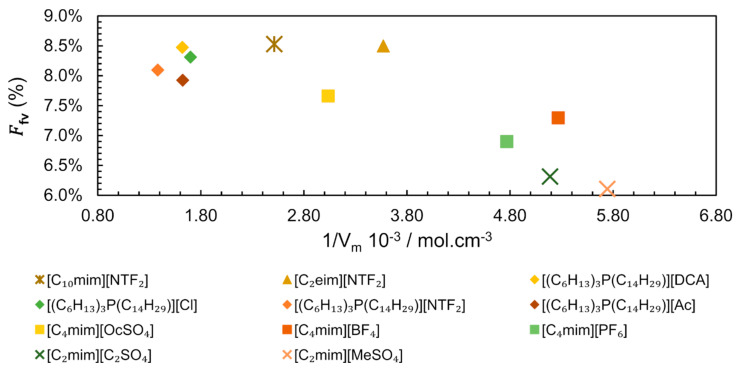
Free volume fraction ($F_{fv}$) dependence of inverse of Molar Volume ($1/V_{m}$ ) of IL at 313 K and 0.1 MPa. The data for trihexyl(tetradecyl)phosphonium-based ILs from references [87,88], [C_4_mim]^+^-based ILs from [89] and the remaining ILs from [90].

Analysing the existing data for different IL families, we constructed a plot correlating $F_{fv}$ versus the inverse of Molar Volume (Figure 14) [87,88,89,90]. The values of $F_{fv}$ vary from 0.0610 for [C_2_mim][MeSO_4_] to 0.0853 for [C_10_mim][NTF_2_], as seen in Figure 14, where $F_{fv}$ is represented as a percentage. Apparently, the points seem scattered showing no correlation between the $F_{fv}$ and the inverse of Molar Volume. However, it is possible to observe that ILs with lower Vm (which corresponds to a higher molar concentration) have a tendency to present lower total free volume, probably due to a more even distribution of smaller ions. A closer look into the structure of the ILs shows that in spite of very different Vm, higher values of $F_{fv}\;$ are more easily reached in ILs with longer linear chains, such as [C_10_mim][NTF_2_] ($F_{fv}$ = 0.0850 and $V_{m}$ = 397.9 cm^3^.mol^−^^1^) and [(C_6_H_1__3_)_3_P(C_1__4_H_2__9_)][NTF_2_] ($F_{fv}$ = 0.0809 and $V_{m}$ = 723.9 cm^3^.mol^−^^1^). 

The effect of molar volumes in the application of ILs was demonstrated by Shannon et al. in CO_2_ capture evaluation [91]. They suggested that the larger the molar volumes of ILs, the higher the CO_2_ solubility, for non-basic anions. However, the molar volume cannot be the only justification, since [C_4_mim][BF_4_] and [C_2_mim][C_2_SO_4_] have similar molar volumes (189.9 and 192.8 cm^3^.mol^−^^1^, receptively), but different CO_2_ solubility, with a CO_2_ molar fraction ($x_{CO2}$) of 0.22 for [C_4_mim][BF_4_] ($F_{fv}$ = 7.3%) and $x_{CO2}$ = 0.11 for [C_2_mim][C_2_SO_4_]($F_{fv}$ = 6.3%), at 313 K and 2.0 MPa [81]. In this case, the solubility of CO_2_ was higher in the IL ([C_4_mim][BF_4_]) with higher $F_{fv}$. Moreover, ILs with more flexible and/or branched chains demonstrated an increase in the solubility of CO_2_, which probably can be related to the presence of available holes with the correct size to accommodate CO_2_ molecules. In other words, the distribution of free volume will influence the solubility of gases and the transport properties of ILs, more than the total free volume [8].

According to the theory of free volume (Equation (8)) developed by Doolittle [92], Cohen and Turnbull [93,94,95,96] and adapted by Macedo [97], the diffusion (*D*) of a molecule/particle in a condensed matter requires two conditions: (i) The occurrence of a cavity greater than a critical size in the vicinity of the diffusing particle; and (ii) This particle having sufficient thermal energy to perform the displacement.

(8) $$D=D_{0}\times exp^{\left(-\frac{\beta \times {a\;}^{*}}{V_{f}}-\frac{{E\;}^{*}}{kT}\right)}$$

where *V_f_* = *a* − a * is the free volume, *E* * is the activation energy, a * is the critical size of the molecular volume, *β* = factor to correct for overlapping free volumes (0.5 to 1.0), *D*_0_, is the Chapman–Enskog self-diffusion coefficient, and *a* = average volume for a molecule in the system.

Beichel et al. [3] have fitted experimental viscosity and conductivity of some [C_4_mim]^+^-based ILs into a similar equation, which ignores the energy requirement for the displacement, Equations (9) and (10).

(9) $$\eta =A_{0}\sqrt{T}\times exp^{\left(\frac{{V\;}^{*}}{V_{l}-{V\;}^{*}}\right)}$$

(10) $$\sigma =\frac{A_{0}}{\sqrt{T}}\times exp^{\left(-\frac{{V\;}^{*}}{V_{l}-{V\;}^{*}}\right)}$$

where σ (conductivity), η (viscosity), $V_{l}$ (volume of liquid, obtained through ρ data) and temperature are the entered values in the equation while $A_{0}$ and ${V\;}^{*}$ are the adjustable fitting parameters (Table 2). ${V\;}^{*}$ represents the critical volume necessary for one molecule to perform the displacement. Through the analysis of the data presented in Table 2, it is possible to observe that the critical volumes (${V\;}^{*}$) obtained with the conductivity (σ) or the viscosity (η) data are similar, but always slightly larger than the sum of the molecular volume of both ions ($V_{Ion\;Pair}$). These results once again demonstrate that there is a dependence on the liquid transport properties of the free volume. However, the model cannot adjust solvents whose molecular structure allows strong interactions by hydrogen bonds, as aqueous solutions, indicating that other transport mechanisms may be predominant in these liquids [98]. 

#### 3.2.2. Experimental Techniques for Free Volume Determination

**Figure 15:**
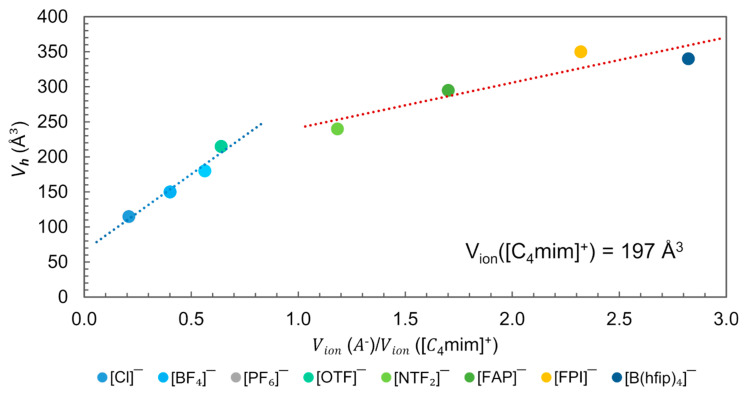
Plot of the hole volume ($V_{h}$) versus Anion–Cation volume ratio $V_{ion}\left(A^{-}\right)/V_{ion}\left(C^{+}\right)\;$ for the [C_4_mim]^+^-based ILs at $T_{k}$. Data from reference [3,88].

Positron Annihilation Lifetime Spectroscopy (PALS) is the most effective experimental method for quantifying the local free volume (hole volume), $V_{h}$ [3]. The technique involves introducing positrons into a sample where they either annihilate on contact with electrons or form metastable bound states, known as positroniums (*Ps*). The lifetime of the trapped positron is sensitively dependent on the size and even the configurational structure of the vacancy [99,100,101]. Beichel et al. related the $V_{h}$ measured with PALS with the ratio $V_{ion}\left(A^{-}\right)/V_{ion}\left(C^{+}\right)$ for [C_4_mim]^+^-based ILs, Figure 15 [3]. The analysis was performed at a temperature $T_{k}$, which denotes the “knee” temperature, above $T_{k}$ the lifetime does not further mirror the true hole size. For larger holes, positron lifetime saturates, and this sets the limit for size dependence [99,100]. Yu et al. observed that $T_{k}$ is close to or even coincides with $T_{m}$ of the corresponding crystalline structure for [C_4_mim][NTF_2_], [C_4_mim][PF_6_] and [C_4_mim][Cl] [101].

In Figure 15, two distinct $V_{h}$ dependencies of the ion size are observed. One for those ILs where the anion–cation volume ratio $\frac{V_{ion}\left(A^{-}\right)}{V_{ion}\left(C^{+}\right)}<1.0$, i.e., the anion has a smaller molecular volume than cation ([C_4_mim]^+^ = 197 Å^3^), and another for anion–cation volume ratio >1.0, when the anion is bigger than the cation. Since for all ILs represented in this figure, $V_{h}$ is smaller than the $V_{ion\;pair}$, and according to the free volume theory, the size of cavities is fundamental for ion displacement, the observed dependence suggests that the displacement of ions in these ILs might be governed by the cation in the first region and by the anion in the second. In the first region, the $V_{h}$ is smaller than $V_{ion}\left(C^{+}\right)\;$ and is greater than the $V_{ion}\left(A^{-}\right)$ when X= [Cl]^−^, [BF_4_]^−^, [PF_6_]^−^. However, in the second region, when X = [FAP]^−^, [FPI]^−^, [B(hfip)_4_]^−^, the $V_{h}$ is greater than the $V_{ion}\left(C^{+}\right)$ and smaller than $V_{ion}\left(A^{-}\right)$. In the ILs with X = [OTF]^−^ and [NTF_2_]^−^, $V_{h}$ exceeds the size of both ions ($V_{ion}\left(A^{-}\right)$ and $V_{ion}\left(C^{+}\right)$).

Despite these differences in the relative size of imidazolium cations and anions and the corresponding $V_{h}$ of the ILs, imidazolium cations generally exhibit larger self-diffusion (*D*) than anions in pure ILs [33]. The explanation obtained by MD simulations shows that the displacement of the imidazolium cation is anisotropic, with the motion normal to the imidazolium ring plane being the most hindered and the motion along the alkyl chain being the most facilitated [102,103]. The magnitude of anion displacement is very similar to cation when the negative ion is moving perpendicular to the imidazolium ring plane or along the direction of the 1-alkyl chain. Thus, the large displacement of cations in ImILs in comparison to anions is mainly due to less hindered dynamics along the direction of the carbon C2 of the imidazolium ring plane. Considering this information, it should be noted that the size of the holes could be smaller than the cation molecular volume. However, only the butyl chain part has a volume (approximately 120 Å) able to move into this neighbouring space, which suggests an initial step for the cation diffusion mechanism [3,82,101].

The Heisenberg spin exchange-dipole–dipole (HSE-DD) separation method on electron paramagnetic resonance (EPR) spectrum was used to measure the translational diffusion coefficients of the ^14^N-labeled perdeuterated 2,2,6,6-tetramethyl-4-oxopiperidine-1-oxyl (^14^N-pDTEMPONE) nitroxide spin probe as a function of temperature in two ImILs series, [C_n_mim]^+^ (n = 2, 4, 6, 8 and 10) with [BF_4_]^−^ and [NTF_2_]^−^ anions [104]. The obtained translational diffusion coefficients of the tracer molecule (^14^N-pDTEMPONE) and IL ions were analysed in terms of the Cohen–Turnbull free volume theory (Equation (8)). The free volume obtained for the nitroxides was compared to the free volume obtained from the self-diffusion of the cations and anions. They noted that even in the cases when the self-diffusion coefficients of the cation and anion were noticeably different, the critical free volumes for the diffusion are the same. In addition, the self-diffusion coefficients of cations (*D*^+^) and anions (*D*^−^) at different temperatures were adjusted to Equation (11) [93,95]. The adjustment parameters for each IL studied are found in Table 3.

(11) $$D=A_{D}\sqrt{T}\times exp^{\left(-\frac{\gamma {V\;}^{*}}{V_{f}^{\prime }}\right)}$$

where D is the self-diffusion, $\gamma {V\;}^{*}$ is the minimum (critical) volume for the diffusion, $V_{f}^{\prime }$ is the free volume per solvent molecule and $A_{D}$ is the adjusting fitting parameter. The authors [104] obtained the values of $V_{f}^{\prime }$ through Equation (12) previously proposed in the literature by Beiche et al. [3] and Yu et al. [82].

(12) $$V_{f}^{\prime }=\frac{M_{w}}{\rho \times Na}-V_{ionpair}$$

where $M_{w}$ is the molar mass, ρ is the density, Na Avogadro number and $V_{Ion\;Pair}$ is the volume of the ion pair (volume of cation plus anion).

**Figure 16:**
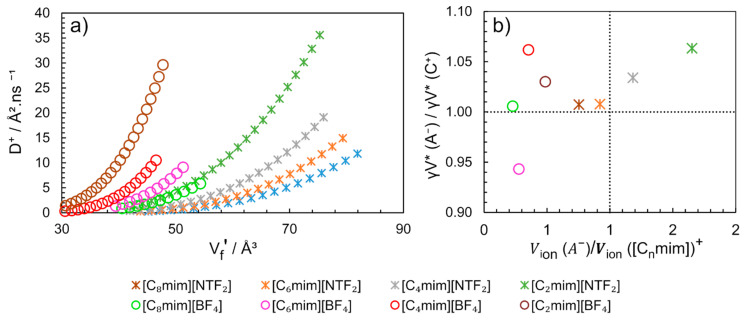
(**a**) Cation self-diffusion (*D*^+^) dependence of free volume per solvent molecule ($V_{f}^{\prime }$) and (**b**) Ratio of critical volume ($\gamma {V\;}^{*}$ ) of the counterions as a function of the ratio of molecular volume of counterions (*V_ion_* (A^−^)/*V_ion_* [C_n_mim]^+^). Graphics constructed from the data published by Merunka and Peric **[104]**, with additional information sent by the authors.

The similarity of the critical free volumes (γV *) obtained for the cation and anion (Table 3) suggests that the diffusion mechanism of the anion and the cation are coordinated. This conclusion is clearly illustrated in Figure 16b, where the ratio between the critical volumes of anion by the cation (γV * (A^−^)/γV * (C^+^)) for different ImILs is close to 1.0 ± 0.06.

When multiplying $V_{f}^{\prime }\times Na$, $V_{f}$ is obtained (cm^3^.mol^−^^1^), using Equations (11) and (12) and the parameters indicated in Table 3, the $V_{f}$ for [C_4_mim][BF_4_] at 313K is equal to 24 cm^3^.mol^−^^1^. Different value has been previously described using the method SL-EoS for the same conditions [89], where $V_{f}$ = 14 cm^3^.mol^−^^1^ was found. However, using the overlap free volumes correction factor in the Equation (11) equal to 0.5, similar values of $V_{f}$ could be obtained by both methods [104]. The $V_{f}^{\prime }$ determined by the authors vary linearly with temperature and present similar values for the same anion family. Additionally, the $V_{f}^{\prime }$ can be correlated with D^+^ (Figure 16a). The diffusion of cation [C_2_mim]^+^ is 50% faster when combined with anion [NTF_2_]^−^ than with [BF_4_]^−^ and the free volume in [C_2_mim][BF_4_] is 50% smaller than in [C_2_mim][NTF_2_].

Therefore, at this point, we can conclude that the volume of the anion is responsible for determining the size of the holes and their distribution in the body of the liquid, and that increasing the number of holes and/or its size will facilitate the molecular transport process. In addition, it is noteworthy to point out that by determining the self-diffusion coefficient (*D*^+^ and *D*^−^), is possible to predict the approximate free volume. These *D*^+^ and *D*^−^ can be directly obtained by NMR by pulsed-field gradient experiments such as diffusion-ordered spectroscopy (DOSY) [105,106]. The technique represents a powerful method in which the cations and anions self-diffusion coefficients can be measured separately and straightforwardly. 

Another technique used to study the local structure of ILs was ^129^Xe NMR spectroscopy. The Xenon atom can occupy cavities in liquids with enough space to accommodate it, [84,107,108,109,110] and the chemical shift (δ) of free ^129^Xe gas, which is close to 0 ppm, can increase up to several hundred ppm for strongly confined environments. Brooks and co-authors [84] have shown a strong correlation between δ and $V_{h}$ ($V_{h}$ obtained from PALS, 10^−9^ s timescale), proving the possibility to use ^129^Xe NMR spectroscopy (10^−3^ s timescale) to study free volume in ILs despite the vastly different timescales involved in the two techniques [84,111]. 

**Figure 17:**
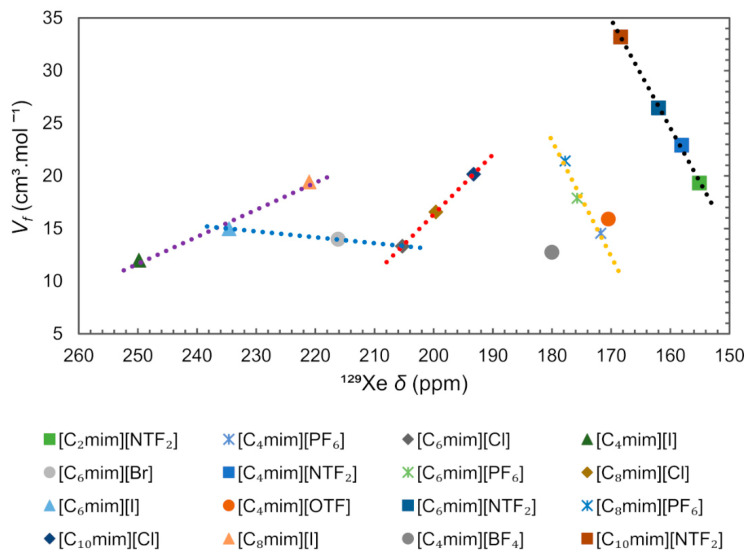
^129^Xe chemical shift (δ) correlated with free volume ($V_{f})$ of ImIL. Dashed lines are only intended to make it easier to identify trends. The δ were taken from reference [108]. The *M* values used to calculate the $V_{f}$ were obtained through the density values for the liquid corresponding to 313 K and 0.1 MPa, from the ILthermo database [112].

In order to check for a possible correlation between free volume and ^129^Xe chemical shift, we compiled the data of ^129^Xe chemical shift for several ImILs, reported by Morgado et al. [108], and related them with the free volume obtained using the equation presented before, the results are presented in Figure 17. It can be concluded that the main ^129^Xe chemical shift differences observed between the described ILs are due to the nature of the anion. The ILs can be grouped according to the type of anion and it is possible to observe five separate trends (dashed lines) between free volume and ^129^Xe chemical shift, depending on the distinct nature of anions, [NTF_2_]^−^ (black), [PF_6_]^−^ (yellow), [Cl]^−^ (red), [I]^−^ (purple), and halogenated anions with [C_6_mim]^+^ cation (blue). Narrower holes in ILs containing halogen anions produce larger ^129^Xe deshielding (>190 ppm) because the average charge distribution around Xe is more positive [110]. Accordingly, the increase in the alkyl chain of [C_n_mim][NTF_2_] and [C_n_mim][PF_6_], moves the ^129^Xe chemical shift to the low field (deshielding). The reverse effect is observed, the ^129^Xe δ moving to a high field (shielding) with increasing alkyl chain, for the [C_n_mim][Cl] and [C_n_mim][I] series. The observed change in chemical shift indicates that the increase in free volume arises within the apolar region of the IL and is likely to consist of an increased number of smaller voids within the liquid structure [107].

In conclusion, with this analysis, it was possible to reinforce the relation between molar volume (Vm), free volume (Vf) and transport properties (such as viscosity, density, diffusivity and conductivity) in ILs systems. As a final remark, considering the high number of studies and applications related to gas solubility in ionic liquid systems, since the size of holes is correlated with the ability of ILs to diffuse, the study of the free volume should be a key property in applications related to gas solubility in these systems.

## 4. Water Effect

When assessing the correlation between structure and physicochemical properties, it is impossible not to consider the effect of the presence of water, since it is an inevitable “contaminant” in real conditions for most organic solvents, including ILs. Two major sources contribute to the presence of water in ILs: it can arise from the IL synthetic route or by absorption due to the hygroscopic character of many ILs. Water is very difficult to remove not only from hygroscopic ILs but also from hydrophobic ILs. As an example, a series of hydrophobic and hydrophilic ILs were exposed to two different moist environments (43 and 81% humidity), and after just five minutes hydrophobic ILs, e.g., [C_4_mim][NTF_2_], absorbed a non-negligible water amount, 0.63 and 1.13 mol%, in 43 and 81% humidity environments, respectively [113].

Insight into the water effect is particularly relevant when aiming to use ILs for widespread applications. At the industrial level, this is one more factor to control, which can be correlated to an increase in the process cost and/or change in operational conditions.

An example of a negative effect of water is in the field of ILs applied to acidic gases (CO_2_ and SO_2_) capture. Some ILs have shown to experience a reduction in the absorption ability with increasing water content [114,115]. However, this hypothesis cannot be generalized since it is IL-dependent, with some ILs-families showing the opposite effect.

The presence of water in ILs is not necessarily a nuisance, and the use as a co-solvent can be helpful to tune some desirable properties to a specific application. The partial water miscibility in some ILs has created an extensive field of application for liquid–liquid separation. Water is practically immiscible in [C_8_mim][BF_4_] at room temperature, but the miscibility increases considerably at higher temperatures. Dyson et al. [116] took advantage of this particularity to separate the catalyst from the products in the 2-butyne-1,4-diol hydrogenation reaction. The reaction was conducted in a water/[C_8_mim][BF_4_] monophasic system at 353 K, with the catalyst and substrates in the same phase. After completion of the hydrogenation reaction, the system was cooled to 283 K where phase separation takes place between the IL and water, with the products in the aqueous phase and the catalyst remaining in the IL phase.

Recently, Yu and Jain [117] reported an elegant strategy for the photochemical conversion of CO_2_ and H_2_O (artificial photosynthesis) to hydrocarbons (C_1_-C_3_) in an aqueous IL medium. They used gold nanoparticles (Au NPs) drop coated in a cotton cloth as catalyst, that was submerged in a [C_2_mim][BF_4_] aqueous solution, in an air-tight vessel that was saturated with CO_2_. The system was then irradiated with green light (incident light beam directed to the substrate containing the nanoparticles) to drive the synthesis of C_1_-C_3_ hydrocarbons from the reduction of CO_2_ and oxidation of water. The excitation of Au NPs yields energetic electron-hole (*e*^−^ − *h*^+^) known to activate the reduction of CO_2_. While water is used as the source of H^+^, the IL is postulated to have a dual role: promotes e– transfer at the interface of the photoexcited Au NP and the adsorbed CO_2_, as well stabilizes the charged radical intermediate formed (CO_2_^−^), increasing its lifetime, making the intermediate more available to proceed with multistep reduction and C-C coupling reaction. The IL promoted reactivity boost precludes the need for an applied potential or an external *h*^+^ scavenger for *e*^−^ − *h*^+^ separation.

The presence of even small amounts of water alters the organizational structure which results in major changes in the IL physicochemical properties, such as viscosity (*η*), density (*ρ*), diffusion (*D*), conductivity (*σ*), as well as inducing, in some cases, the formation of nanodomains [118] and nanoclusters [119]. The implications of water content in ILs physicochemical properties have been the subject of several studies, concerning interactions, dynamics, transport properties and microstructure, which have been compiled in a very scarce number of reviews, usually focused on one physicochemical property or an application [120,121,122,123,124,125]. These implications can be divided into three main sub-subjects: water in the bulk, at the interface and in confinement, with most of the reports on the first topic.

**Figure 18:**
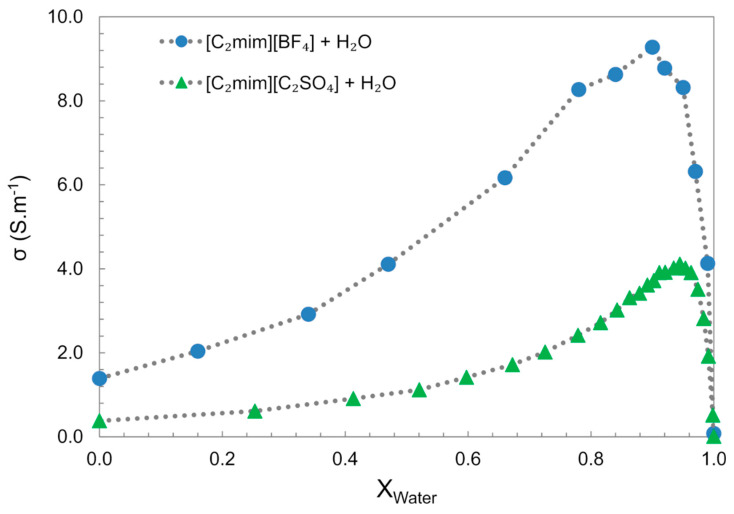
Electrical conductivity (*σ*) of [C_2_mim][C_2_SO_4_] + water and [C_2_mim][BF_4_] + water at 298 K. Data from references [61,126].

In the bulk water, the electrical conductivity generally increases with increasing water concentration, until it reaches a maximum value at considerable high IL dilution (~0.9 x_w_), followed by a decrease to values similar to “pure water”, Figure 18 [61,126]. This behaviour is divergent from aqueous salts solutions, e.g., NaCl and NaOH, which show an almost linear electrical conductivity decrease with the increase in water content. The increase in electrical conductivity with the increase in water content can be particularly explained by two factors: the decrease in the mixture viscosity allows higher ion mobility; the other is the segregation of water molecules into clusters that allow the ions to move freely. The conductivity decrease observed in the water-rich domain results from the dispersion of the charge carriers in the media.

**Figure 19:**
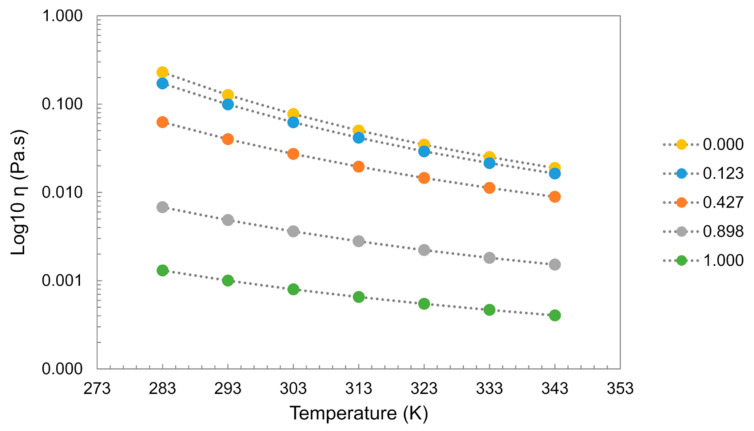
Mixture of [C_2_mim][C_2_SO_4_] + H_2_O. Viscosity (η) dependence of temperature (T) and water molar fraction (X_w_). Data from references [59,60,127].

Viscosity is very sensitive to impurities in the system, with ILs showing tremendous viscosity changes even in the presence of small amounts of water. In most cases, water addition induces a decrease in the viscosity of the system. As an example, for [C_2_mim][C_2_SO_4_], the addition of water induces a decrease in the viscosity showing a greater impact at lower temperatures as depicted in Figure 19 [59,60,127].

However, there are some exceptions and [C_4_mim][Ac] viscosity shows a water content viscosity dependency. Initially, a small water amount (up to 0.02 wt%) induces an increase in the viscosity, which decreases with the increase in water addition [128]. By MD simulations, the authors attributed this anomalous behaviour to a water-induced structural change in the IL, with the formation of chain-like {anion•••water•••anion•••}n structure, which length increases with the water content up to 0.02 wt%. In terms of interactions, there is a hydrogen bond (HB) weakening between ion pairs but the overall balance shows an increase in the global HB due to water/anion interactions. This phenomenon was not observed for [C_4_mim][BF_4_] and [C_4_mim][NTF_2_], which by comparative structural analysis between [C_4_mim][Ac] and [C_4_mim][BF_4_] showed the latter forming a more spherical-type complex, which has higher mobility than the chain structure.

Several experimental methods have been applied to study the structure and dynamics of both neat IL and its mixtures with molecular solvents, particularly for phase-behaviour of water-IL mixtures, from DFT calculations [129,130,131] and molecular dynamics (MD) simulations [132,133,134], to spectroscopic techniques like NMR [32,106,135,136,137,138], Small-Angle Neutron Scattering (SANS) [139,140], infrared and RAMAN spectroscopy [141,142,143,144,145].

MD simulation has been one of the most widely used and has assumed an essential role in understanding the molecular interactions in ILs, particularly the anomalous phenomena observed in the macroscopic properties both for neat and IL-molecular solvent mixtures. Since the early work by the Lynden-Bell group [146,147] and also the Maginn group [148,149] several theoretical studies have been reported for ILs, both in neat and as molecular solvents mixtures, see for instance reviews [32,120,121,132,150,151] and book chapter [152] and references therein. It is important to highlight that most of the work has been focused on imidazolium-based ILs, with preponderance for [BF_4_]^−^ and [PF_6_]^−^ anions. 

One of the earliest studies for IL-water mixtures by Hanke and Lynden-Bell, compared a hydrophilic ([C_1_mim]Cl and a hydrophobic ([C_1_mim][PF_6_]) IL. They showed that at low water concentrations the water molecules are isolated or in small clusters [146]. Above 75% water molar ratio, there is a combination of percolating water, small clusters and isolated water. The main differences between the systems were on the signal of the excess volume, and the molecular motion behaviour (both rotational and translational), however the motion for both ILs becomes faster with the increasing amount of water. By Far-infrared (FIR) spectroscopy, Dominguez-Vidal et al. [143] showed for [C_2_mim][BF_4_], [C_4_mim][BF_4_] and [C_4_mim][PF_6_] water mixtures, at different concentrations, that water molecules bind more strongly to [BF_4_]^−^-based ILs (hydrophilic) than to [PF_6_]^−^ (hydrophobic). The interaction between monomeric water and the anions is absent in [C_4_mim][PF_6_] aqueous solution IR spectra, indicating that the water/[PF_6_]^−^ interaction is very weak, which is in agreement with DFT calculations that showed that [PF_6_]^−^ anions could not form stable complexes with water, opposite to [BF_4_]^−^ [153].

**Figure 20:**
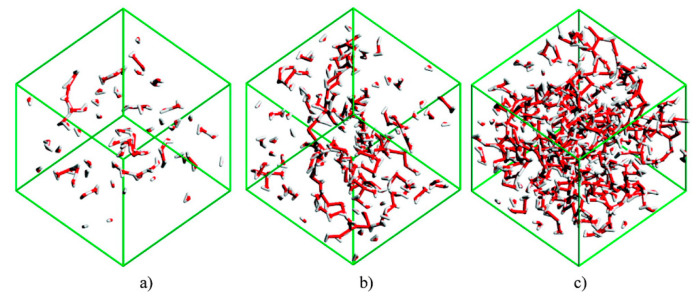
Snapshot images of the water clusters in simulation boxes with (**a**) $x_{H_{2}O}=0.5$, (**b**) $x_{H_{2}O}=0.8$, and (**c**) $x_{H_{2}O}=0.92$. Fully reproduced image from reference [154].

Bernardes et al. [154] studied [C_2_mim][C_2_SO_4_] + H_2_O system and identified four distinct solution regimes (Figure 20):

For $x_{H_{2}O}$ ≤ 0.5, water molecules are either isolated or in small chain-like clusters;

For 0.5 < $x_{H_{2}O}$ < 0.8, the water molecules are surrounded by two or less water molecules, suggesting the formation of linear chains. These chains grow and start to entangle the IL polar network; 

For 0.8 < $x_{H_{2}O}$ <between 0.8 and 0.95, the water molecules transition from a chain-like aggregate to a 3D web network, forming with the IL two distinct continuous networks;

$x_{H_{2}O}$ < 0.95, the polar network of the IL starts to break into smaller aggregates and loses its continuous nature. Water molecules begin to experience levels of connectivity similar to that found for pure water.

Trinidad Méndez-Morales et al. [155] performed extensive MD simulations to investigate the structure and some dynamic properties of aqueous mixtures of the hydrophobic family [C_n_mim][PF_6_] and hydrophilic [C_n_mim][Cl] and [C_n_mim][Br] (*n* = 2, 4, 6 or 8). The authors stated that water tends to cluster in the IL cavities inside the IL polar domains, in which the structuring role of water in the system strongly depends on the hydrophobicity degree of both the cation and the anion, but most strongly from the latter, showing a higher degree of water clustering with the increase in the hydrophobic character of the system [155,156]. These water clusters were labelled as “water-pocket” or “confined water” (non-bulk water) into the IL framework. At low water content, most of the macroscopic properties are virtually unaffected, with a slight increase in the self-diffusion and a decrease in the viscosity.

Extensive experiments have been carried out to probe the existence of water pockets. Small-angle neutron scattering (SANS) measurements enable the analysis of the distribution of deuterated water between being molecularly dissolved and microphase separated, upon mixture with ILs.

Abe et al. [157,158] used a complementary methodology of SANS and Small-angle X-ray scattering (SAXS) to study the water-rich regions of [C_4_mim][NO_3_], [DEME][BF_4_] and [DEME][NO_3_] (DEME=*N*,*N*-diethyl-*N*-methyl-*N*-(2-methoxyethyl) ammonium) aqueous solutions, 70–90 mol % D_2_O. For the imidazolium-based IL, they identified the presence of water pockets, with an average size of 20 Å, which were not present in [DEME][BF_4_] and [DEME][NO_3_], suggesting that cation interactions can be responsible in the control of water pocket formation.

**Figure 21:**
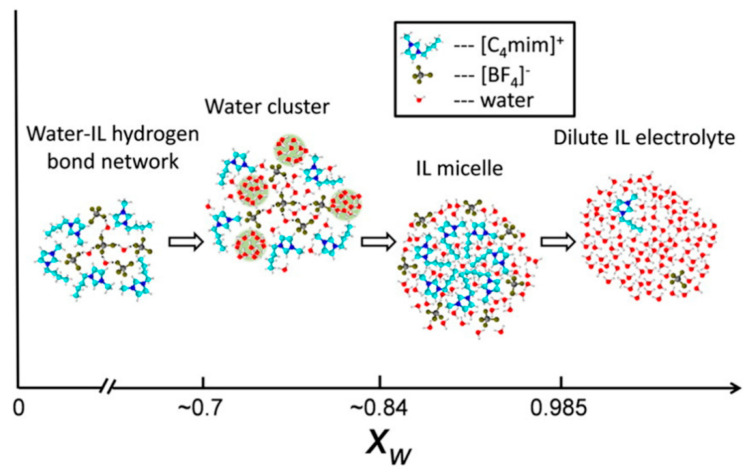
Schematic illustration of structures in mixtures of [C_4_mim][BF_4_] and D_2_O determined by small-angle neutron scattering (SANS), fully replicated image from reference [119].

Focusing on the hydrophilic IL [C_4_mim][BF_4_], Gao and Wagner [119] investigated the microstructure of the IL rich region of IL/deuterated water mixture by SANS. The authors claimed the existence of three water content domains (Figure 21). Up to 2:1 water:IL mol ratio (x_w_ = 0.66), the water molecules are lodged in the IL polar network, interacting with cations and anions by hydrogen bonds, without any significant change in the microstructure of the IL. Further addition of water x_w_~0.7 results in the formation of clusters, whose size increases with the addition of D_2_O, forming a partition between water in the dissolved state and a microphase separated state. Above ~80% mol, a phase inversion occurs, forming IL aggregates.

**Figure 22:**
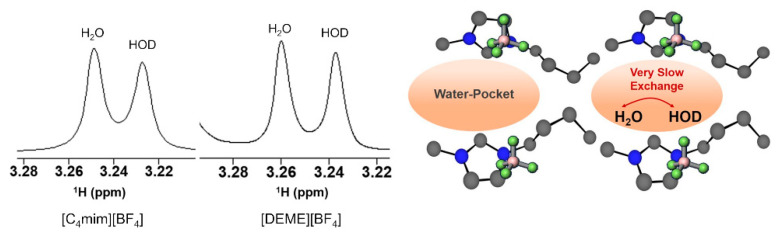
^1^H NMR spectra of 50 mol% water mixtures of [C_4_mim][BF_4_] and [DEME][BF_4_]. Schematic representation of the “two waters” in [C_4_mim][BF_4_]-50 mol % water, from reference [118].

The properties of water confined in [C_4_mim][BF_4_] have also been investigated by NMR. Saihara and co-authors [118] followed up the SAXS and SANS studies reported by the same research group (vide Abe et al. described above [157]), using partially deuterated water (HOD, a 1:1 mixture of H_2_O and D_2_O) as a probe to explore the state of the water molecules inside the IL framework in a water/IL mixture (IL = [C_4_mim][BF_4_] and [DEME][BF_4_]). The authors acquired ^1^H NMR spectra of IL + partially deuterated water mixtures (50 mol% water) (Figure 22), which showed that the water signals split into two distinct peaks, corresponding to HOD and H_2_O. In solution, the ^1^H NMR spectrum of a mixture of H_2_O:D_2_O (1:1) shows only one peak due to the fast H/D exchange, hence the presence of IL decreases the exchange rate among the water molecules in the IL to a very slow exchange regime. The authors attributed this slowing down to the water experiencing different environments because of water molecules being confined inside IL nanodomains (“water pocket”).

For [DEME][BF_4_] the authors observed the same behaviour in the ^1^H NMR spectra, even further changing the partially deuterated water composition in the mixture to 20–90% mol, the split signals are observed from the lowest water content up to 80% mol. [DEME][BF_4_] has previously been analysed by SANS/SAXS [157,158] with no evidence of water pocket presence, which contradicts the affirmation by Saihara et al. that HOD/H_2_O peak splitting is an indicator for the existence of “water pockets”. This inconsistency was recently pointed out by Bystrov et al. [138], who also added that the presence of split signal from low water content up to 80% mol contradicts the previous work that stated the presence of water pockets between 70–90% mol fraction. Additionally, the SANS data show that the water pockets should have a considerable size (average 30 Å), which corresponds to almost 1000 water molecules, that would be organized in a 3D framework. To obey the slow exchange regime condition, $2\pi \Delta \upsilon t_{exc}\gg 1$ (where Δυ is the linewidth variation between the peaks (Hz) and $t_{exc}$ is the exchange time) the exchange time should be higher than 200 ms, which would be improbable in a 3D structure inside the pocket (pure water exchange time is ~1 ms).

**Figure 23:**
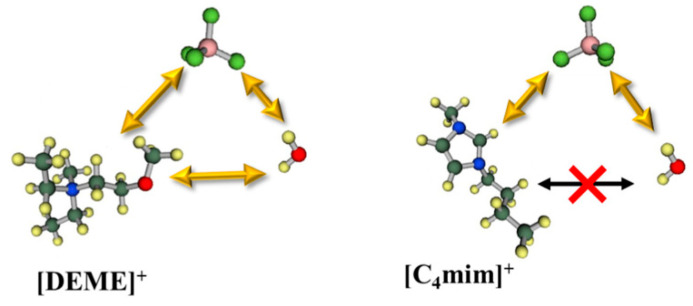
Molecular interactions of [DEME][BF_4_]–H_2_O and [C_4_mim][BF_4_]–H_2_O. Image fully reproduced from reference [139].

In a recent paper by Abe et al. [139], the cation effect in the formation of a water pocket was clarified comparing [C_4_mim][NO_3_] with [DEME][NO_3_] water mixtures. SANS shows only evidence of “water pockets” formation in the imidazole system. Previous [C_4_mim][BF_4_]-water MD simulations showed that molecular interactions between the cation and water molecules have a repulsive nature, which the authors suggested to be the driving force to exclude the water molecules, favouring the existence of “water pockets” (Figure 23). For the [DEME]^+^ cation, its structure has an ether group, which would interact with water molecules, that migrate between cation and anion, making it very unlikely to form pockets. These data are in agreement with the inconsistencies noted by Bystrov and co-authors [138] on the conclusions by Saihara and co-authors [118]. Hence, the signal splitting of the HOD/H_2_O ^1^H NMR chemical shift could explain water pockets, however, it should not exist in [DEME]-IL system. SANS show no water pocket formation and cannot explain the slow-exchange between the different waters.

The self-diffusion coefficients can be used to probe the phase behaviour in ILs, both neat and for mixtures. Cascão et al. [106] studied [C_4_mim][BF_4_] + H_2_O system by multinuclear NMR approach, to probe in detail the translational and rotational motion of the cation and anion at different water content. The authors identified a critical water composition of 10% mol water, where the cation and anion share the same self-diffusion coefficients (and therefore share the same hydrodynamic radius) which indicates the presence of a special kind of aggregation or long-lived ion pair/water cluster. Water at this composition has a higher effect on the rotational diffusion of the anion, showing a sharp decrease to values similar to diluted IL. These data suggested that water could be incorporated in the IL nanostructure, allowing water molecules to position close to the cation, competing with the anion to establish interactions with the cation. This leads to the disruption of some cation/anion interactions increasing both the rotational and translational dynamics of the anion.

By MD simulations, Moreno and co-authors [159] studied [C_4_mim][BF_4_] and its mixtures with water, concluding that the relative distance of the anion is closer to cations’ H2 (between the two nitrogens of the imidazolium ring) and water molecules, and further away from [C_4_mim]^+^ aliphatic chain. The authors also concluded that by increasing the water concentration, the anions are more dispersed in the aqueous medium than the cations, with the stability of the ionic pair beginning to be affected at x_w_ = 0.20.

**Figure 24:**
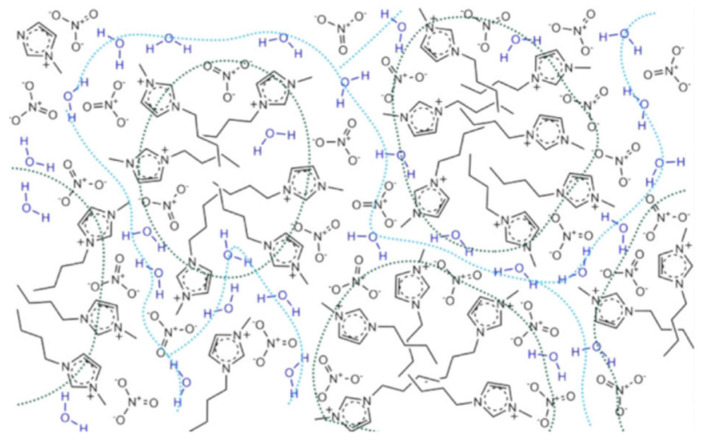
Model proposed by Bystrov et al. [138] of a mixture of IL and water (x_w_ ≈ 50% mol) The black dotted lines indicate hydrophobic fragments, and the blue ones show the “facilitated” diffusion paths of water molecules. Image edited from [138].

Bystrov et al. [138] also studied the self-diffusion by pulsed-field gradient-stimulated echo (PFGSTE) NMR and relaxation rates in a set of imidazolium-based ILs ([C_4_mim][X], [X] = [BF_4_]^−^, [NO_3_]^−^, [OTF]^−^, [Cl]^−^, [Br]^−^ and [I]^−^) and their aqueous mixtures. The combination of the experimental NMR data with MD simulation studies led the authors to disprove the water pocket model proposed by Saihara, considering as alternative a model where the water molecules are dispersed (“spread”) along with the hydrophilic domains of the IL, which could be applied up to 80–90% water concentration. This model was created based on the well-established heterogeneity of neat ILs, that presents the distribution of polar and non-polar domains, in which the water molecules are dispersed in crevices surrounding the hydrophilic regions of the cations or, mainly, near the anions (Figure 24). This model is very similar to that proposed by Rollet et al. [160] for [C_4_mim][NTF_2_] aqueous mixtures based also in PFGSTE NMR, in which the water molecules form thin “streams”/”creeks” as the water concentration increases, with partial segregation between more IL and less IL enriched phases.

As seen by the examples discussed above, the question of how the addition of water correlates to the structural modifications is very complex and instigates a heated scientific debate. Even very recently, two papers, Verma et al. [133] using MD simulations with ab-initio force fields, and Yoshimura et al. [142] using NMR and Raman spectroscopy, formulated a new model for [C_4_mim][BF_4_]/water systems at low water content. Both works maintained the idea that at low water content the water is “trapped” inside the IL network, with small water clusters characterized by water molecules with three or four hydrogen bonds per molecule, indicating that local structure approaches bulk water like coordination environment. 

While the amount of experimental data gathered is remarkable, the interpretation has been at least very subjective as seen by the different available models. Although the concept of designing a global model for IL-water mixtures towards the tuning of physicochemical properties for a given application would be a major achievement, this seems a herculean task even focusing on only an IL family. 

## 5. Conclusions

Almost 30 years of research on ILs was presented from the perspective of transport properties and their relationship with structure, organization, size and volume.

The local nanostructural organization and the physicochemical properties of ILs are directly related. The intermolecular interactions present in the system are determined by the ion structure and can have a sophisticated organization that is ultimately the result of the balance of the many types of interaction possible between the ions (hydrogen bonds, Van der Waals, etc.). These will determine the interionic distances and structural heterogeneity, and consequently, the existence of aggregates, ion pairs and the free volume.

The phenomenon of ion-pair formation in ILs was addressed and the different definitions of ion-pairs were reviewed. Despite the different definitions that mainly differ in the lifetime and dynamics/identity of the interacting counter ions, it was shown that the transport properties are strongly influenced by the rate of association/disruption of these close interacting cation and anion species.

The importance of the molecular volume (volume of ions) as the main determinant of IL transport properties (viscosity, conductivity, diffusivity) was highlighted and its relation to the free volume was described. It is possible to predict the density, viscosity and conductivity of families of ILs as a function of the molecular volume and the anion type. The volume of the ions and their nature determine the size of the free volume and the way it is distributed inside the liquid (hole volume, $V_{h}$). 

Several methodologies to estimate IL free volume were reviewed. The free volume can be estimated from the Sanchez–Lacombe equation of state, through the pressure–temperature density dependence. It can also be obtained using the density and molecular volume of the IL (obtained through crystallographic or scaled data). Experimental techniques such as Positron Annihilation Lifetime Spectroscopy can be used to measure/inform the average volume of holes, but is not able to measure the quantity (distribution) of holes and consequently the free volume.

The Cohen and Turnbull equations have been used successfully to correlate transport properties to the free volume of ILs. This has been known for almost five decades for non-functionalized organic solvents.

In summary, from the literature reviewed here and our own findings, we can highlight the following points:

1. The free volume ($V_{f}$) of ionic liquids increases with temperature (isobaric expansion) and decreases with pressure (isothermal compression);
2. The molar volume (Vm) of ILs is associated with the free volume;
3. The free volume is distributed in holes/voids;
4. The size of holes is correlated with the ability of ILs to diffuse and to solubilize gases;
5. The nature of the cation–anion interaction will determine how hole volume ($V_{h}$), free volume ($V_{f}$) and the ion pair volume ($V_{fionpair}$) will influence the transport properties of liquids.

The water effect in ILs was lightly reviewed in this paper, selecting examples that address the peculiar interactions present in aqueous ILs. Overall, the effect of water in ILs can be summarized into four main regions:

1. Low water content (water in IL; x_H_2_O_ < 0.1) regime; the water molecules are dispersed in the IL nanostructure, filling the structural holes present in the ILs 3D network, without disrupting it;
2. Intermediate regime (0.2 < x_H_2_O_ < 0.8). Increasing the IL dilution, water starts to aggregate, forming clusters. While the formation of clusters has been accepted by the majority of studies, the shape, size and morphology are still subject to discussion, in which the formation of water “pockets” and creek like structures (water dispersion in IL) are the two main hypotheses, connecting polar domains of the IL;
3. High water regime (IL in water; 0.8 < x_H_2_O_ < 0.9). The clusters are partially or totally destroyed, with the ions experiencing a solvent-mediated ion pair, leading to increased spatial associations between ions and, thus, having a strong effect on translational and rotational dynamics of ion species in mixtures;
4. Diluted solutions (x_H_2_O_ > 0.95). Above this water content, there is an infinite dilution of ILs in water, with the ions fully hydrated, forming a loose ion pair, with physicochemical properties similar to pure water.
5. While the topics of this review have been addressed separately, they cannot be dissociated from one another. In the case of aqueous IL solutions, the heterogeneity and the clustering of water not only affects the physicochemical properties and dynamics but can also be rationalized as a function of the structural organization.

Overall, IL properties were shown to depend on the molecular scale details of structure and dynamics, which are reflected in the intermolecular interactions and can be traced back to aggregate formation, ion-pairing and free volume.

## Figures and Tables

**Table 1:** Molecular volume of ion pair (V_IonPair_) of ILs separated by anionic group, data from Reference [79].

Entry	[Anion]^−^ Group	[Cation]^+^	V_ionpair_ (nm^3^) ^[a]^
1	[NTF_2_]	[C_2_mim]	0.388
2		[C_3_mim]	0.410
3		[C_4_mim]	0.428
4		[C_5_mim]	0.451
5		[C_4_mmim]	0.461
6		[C_4_NMe_3_]	0.430
7		[C_4_mpyr]	0.453
8		[C_5_mpyr]	0.470
9		[C_5_NEt_3_]	0.500
10		[SEt_3_]	0.409
11		[bpy]	0.430
12		[MeSPh_2_]	0.500
13	[BF_4_]	[C_4_mim]	0.269
14		[C_6_mim]	0.315
15		[C_8_mim]	0.361
16	[PF_6_]	[C_4_mim]	0.305
17		[C_6_mim]	0.351
18		[C_8_mim]	0.397
19	[DCA]	[C_2_mim]	0.240
20		[C_4_mim]	0.267
21		[C_4_mpyr]	0.295

**^[a]^** The ionic volume was determined from crystal structures containing the ion of interest in combination with a reference ion of known volume.

**Table 2:** Fit parameters $A_{0}$ and ${V\;}^{*}$ for viscosity (η) and conductivity (σ) of [C_4_mim][X], according to Equations (9) and (10). Data from reference [3].

Entry	[X]^−^	$V_{IonPair}$ $\;({Å}^{3}){\;}^{\left[a\right]}$	$A_{0}$ ${\;}^{\left[b\right]}\;(10^{-7}\;\mathrm{Pa}\cdot s\cdot K^{-0.5})$	${V\;}^{*}$ $\;({Å}^{3}){\;}^{\left[b\right]}$	$A_{0}$ ${\;}^{\left[c\right]}\;(10^{4}\;\mathrm{mS}\cdot {\mathrm{cm}}^{-1}\cdot K^{-0.5})$	${V\;}^{*}$ $\;({Å}^{3}){\;}^{\left[c\right]}$
1	[Cl]	238	2.50	253	69.8	251
2	[BF_4_]	276	9.88	281	9.8	274
3	[PF_6_]	308	10.56	315	9.7	308
4	[OTF]	323	6.13	333	8.0	324
5	[NTF_2_]	430	7.16	432	6.4	423
6	[B(hfip)_4_]	753	15.44	879	0.8	839

^[a]^ The value of $V_{IonPair}$ the authors took from crystallographic data, if available in the literature, or scaled it from similar structures [3]. ^[b]^ Obtained from η. ^[c]^ Obtained from σ.

**Table 3:** Fit parameters, $A_{D}$ and γV *, for the diffusion coefficients of cations, D^+^, and anions, D^−^, in ILs according to the Equation (12), and the determined $V_{f}^{\prime }$ at 313 K and 1 bar, data from reference [104].

Entry	ILs	$V_{f}^{\prime }/{Å}^{3}$	$A_{D^{+}}/{Å}^{2}\cdot {\mathrm{ns}}^{-1}$	γV * ^[a]^/Å^3^	$A_{D^{-}}/{Å}^{2}\cdot {\mathrm{ns}}^{-1}$	γV * ^[b]^/Å^3^
1	[C_2_mim][BF_4_]	41.8	264 ± 20	245.9 ± 3.4	272 ± 21	253.3 ± 3.5
2	[C_4_mim][BF_4_]	39.1	371 ± 35	302.4 ± 4.2	579 ± 47	321.1 ± 3.6
3	[C_6_mim][BF_4_]	39.4	214 ± 14	313.2 ± 3.2	153.1 ± 7.2	295.4 ± 2.2
4	[C_8_mim][BF_4_]	40.6	68.9 ± 0.9	294.4 ± 0.6	85.4 ± 1.6	296.1 ± 0.9
5	[C_2_mim][NTF_2_]	58.0	94.6 ± 3.6	296.6 ± 2.6	74.9 ± 2.4	315.4 ± 2.2
6	[C_4_mim][NTF_2_]	59.6	103.5 ± 4.8	352.3 ± 3.2	97.6 ± 4.1	364.3 ± 3.0
7	[C_6_mim][NTF_2_]	60.7	83.3 ± 2.1	370.4 ± 1.8	75.8 ± 2.1	373.3 ± 2.0
8	[C_8_mim][NTF_2_]	61.2	57.2 ± 0.5	370.6 ± 0.7	57.1 ± 0.5	373.3 ± 0.6

^[a]^ Cation analysis. ^[b]^ Anion analysis.

## Abbreviations

**Symbols**	
$\alpha_{p}$	isobaric expansivity
δ	chemical shift
η	viscosity
ρ	density
σ	conductivity
D	self-diffusion
$F_{fv}$	free volume fraction
I	ionicity
$K_{T}$	isothermal compressibility
$M_{w}$	molecular weight
M	molar concentration
$N_{A}$	Avogadro number
$T_{c}$	crystallization temperature
$T_{g}$	glass transition
$T_{m}$	melting point
$V_{h}$	volume of holes
$V_{IonPair}$	molecular volume of the ion pair
$V_{c}$	volume of crystal
$V_{counterion}$	volume of the counterion
$V_{i}$	volume interstitial
$V_{f}$	free volume
$V_{ion}A^{-}$	volume molecular of anion
$V_{ion}C^{+}$	volume molecular of cation
$V_{l}$	volume of liquid
Vm	volume molar
$V_{occ}$	volume occupied
$V_{w}$	Van der Waals volume
x	molar fraction
**General Abbreviations**	
APIL	Aprotic ionic liquid
API	*Active Pharmaceutical Ingredients*
COSMO-RS	Conductor-like Screening Model for Real Solvents
DFT	Density Functional Theory
*E*_HB_	hydrogen-bonding interaction energy
HB	hydrogen bond
EoS	equation of state
EPR	electron paramagnetic resonance
Eq	equation
FIR	Far-infrared
HSE-DD	Heisenberg spin exchange-dipole–dipole
IC	ionic cage
IL	ionic liquid
ImIL	imidazolium-based ionic liquid
IP	ion pair
MD	molecular dynamics
NP	nanoparticles
PALS	Positron Annihilation Lifetime Spectroscopy
PGSE	pulse-field-gradient spin–echo
PIL	Protic ionic liquid
PCM	polarizable continuum model
Ps	positroniums
pDTEMPONE	2,2,6,6-tetramethyl-4-oxopiperidine-1-oxyl
QENS	quasielastic neutron scattering
SL	Sanchez–Lacombe
SANS	Small angle neutron scattering
SAXS	Small angle X-ray scattering
VdW	Van der Waals
**Ionic liquids acronym**	
[Ac]^−^	acetate
[BETI]^−^	bis(pentafluoroethylsulfonyl)imide)
[BF_4_]^−^	tetrafluoroborate
[B(hfip)_4_]^−^	1,1,1,3,3,3-hexafluoropropan-2-yl)oxy)borate
[BPy]^+^	*N*-butylpyridinium
[C_2_NH_2_]^+^	diethylammonium
[C_2_SO_4_]^−^	ethylsulfate
[C_2_eim]^+^	1,3-diethylimidazolium
[C_2_mim]^+^	1-ethyl-3-methylimidazolium
[C_3_mim]^+^	1-propyl-3-methylimidazolium
[C_4_mim]^+^	1-butyl-3-methylimidazolium
[C_12_mim]^+^	1-dodecyl-3-methylimidazolium
[C_4_mmim]^+^	1-butyl-2,3-dimethylimidazolium
[C_4_mpyr]^+^	*N*-butyl-*N*-methylpyrrolidinium
[C_4_NMe_3_]^+^	butyltrimethylammonium
[C_5_mpyr]^+^	*N*-pentyl-*N*-methylpyrrolidinium
[C_5_NEt_3_]^+^	pentyltriethylammonium
[C_6_mim]^+^	1-hexyl-3-methylimidazolium
[C_8_mim]^+^	1-octyl-3-methylimidazolium
[(C_6_H_13_)_3_P(C_14_H_29_)]^+^	trihexyltetradecylphosphonium
[CF_3_CO_2_]^−^	trifluoroacetate
[DCA]^−^	dicyanamide
[DEME]^+^	*N*,*N*-diethyl-*N*-methyl-*N*-(2-methoxyethyl) ammonium
[EtNH_3_]^+^	ethylammonium
[FAP]^−^	tris(perfluoroalkyl)trifluorophosphate
[FPI]^−^	bis[bis(pentafluoroethyl)phosphinyl]imide
[NTF_2_]^−^	bis(trifluoromethylsulfonyl)Imide
[MeSPh_2_]^+^	methyldiphenylsulfonium
[MeSO_4_]^−^	methylsulfate
[NO_3_]^−^	nitrate
[OcSO_4_]^−^	octyl sulfate
[OTF]^−^	triflate
[PF_6_]^−^	hexafluorophosphate
[SEt_3_]^+^	triethylsulfonium
